# Digitally delivered full plate living: associations with diet quality, body mass index, and participant-reported health measures among participants with paired assessments

**DOI:** 10.3389/fdgth.2026.1947833

**Published:** 2026-10-12

**Authors:** Rebecca K. Kelly, Amy Hanus, Janet Calhoun, Jonathan P. Bonnet, Ron Stout

**Affiliations:** 1 Element Health, Inc., Fairhope, AL, United States; 2 Ardmore Institute of Health, Ardmore, OK, United States; 3 Stanford University School of Medicine, Stanford, CA, United States

**Keywords:** digital nutrition, full plate diet, health improvement, plant-predominant, weight change, whole food, body mass index, diet quality

## Abstract

**Introduction:**

Nutrition counseling is often limited by time, staffing, and resources. Full Plate Living (FPL) is a free, self-guided digital nutrition program that encourages a whole food, plant predominant, fiber-rich eating pattern. This study examined baseline-to-follow-up changes in diet quality, body mass index (BMI), dietary fiber intake, and participant-reported health measures among adults with eligible paired Diet ID/FPL assessments.

**Methods:**

This secondary analysis used de-identified observational records from July 2023 through January 2026. Baseline was the first available record, and follow-up was the first subsequent record at least 60 days later. Baseline characteristics were compared between participants with and without eligible paired records. Within-participant changes were evaluated using paired analyses, with Bonferroni-adjusted sensitivity analyses for multiple outcomes. Regression analyses examined whether follow-up interval was associated with outcome change.

**Results:**

Among 4,359 participants with 7,077 records, 392 (9.0%) had eligible paired records. The paired-assessment cohort was predominantly female (87.5%), White (81.1%), mean age was 57.7 years. Compared with participants without eligible follow-up records, paired-assessment participants were older and had higher diet quality and dietary fiber intake and lower estimated energy intake. Among participants with paired data, Diet ID Quality Score increased from 7.20 to 7.91 (Cohen d=0.26), Healthy Eating Index-2015 increased from 73.87 to 80.24 (d=0.27), BMI decreased from 31.62 to 31.03 kg/m² (d=−0.17), and dietary fiber intake increased by 5.0 g/d (d=0.28). After Bonferroni adjustment, improvements in diet quality, healthy food choice confidence, weight-loss confidence, energy level, general health, dietary fiber intake, and food group measures remained significant, along with the modest BMI reduction; sleep duration category did not remain significant. Longer follow-up interval was weakly associated with smaller improvements in Healthy Eating Index-2015 and dietary fiber intake and was not associated with BMI change. DISCUSSION: Among participants with eligible paired assessments, baseline-to-follow-up improvements were observed in diet quality, dietary fiber intake, participant-reported health measures, food group measures, and BMI; effect sizes were small. These findings should be interpreted as exploratory repeated-observation changes rather than causal evidence of program effectiveness. These findings may guide evaluation of FPL as a no-cost digital nutrition resource for individuals, clinics, and communities.

## Introduction

Chronic diseases, including cardiovascular disease, type 2 diabetes, obesity, and certain cancers, remain leading causes of morbidity and mortality in the United States and globally (1). Lifestyle related factors, including diet quality, physical activity, sleep, avoidance of alcohol, tobacco and substances, weight management, and cardiometabolic risk-factor control, are central elements for chronic disease prevention, lifestyle medicine, and whole person care (2–4). Primary care providers and other health care professionals are well positioned to identify diet related chronic disease risk and initiate lifestyle medicine conversations; however, routine clinical practice often provides limited time and infrastructure for intensive nutrition counseling. Barriers include competing visit priorities, reimbursement limitations, variable nutrition training, limited referral pathways, patient access challenges, and inconsistent follow-up (5, 6). Patients may receive brief advice to improve diet quality, increase intake of fruits and vegetables, lose weight, or manage cardiometabolic risk, but many need practical, accessible, and ongoing support to translate these recommendations into daily food choices between clinical encounters.

Digital health interventions may increase the reach of lifestyle medicine providers, clinics, and communities by providing flexible access to education, self-monitoring tools, behavior-change tools, and supportive resources between clinical visits (7). Digital nudging has been evaluated as one approach to improving dietary quality in randomized trials (8). However, sustained dietary behavior changes remain difficult in real-world settings. Food access, time constraints, cost, limited cooking experience, social norms, competing family and work demands, and low confidence can all affect long-term adherence (9). These barriers underscore the need for practical, low-cost, scalable nutrition resources that can be used across clinical and community settings.

Whole food, plant-predominant, fiber-rich dietary patterns have been associated with favorable metabolic, weight-related, cardiovascular, cancer-related, and mortality outcomes in observational studies, systematic reviews, and meta-analyses (10–13). Higher overall diet quality, as measured by indices such as the Healthy Eating Index-2015 (HEI-2015), has also been associated with lower risk of all-cause, cardiovascular disease-related, and cancer-related mortality (14, 15). The HEI framework is widely used to measure consistency with federal dietary guidance and has been updated over time to reflect changes in the Dietary Guidelines for Americans (16). Psychosocial context is also relevant to lifestyle medicine, as social isolation and loneliness have been associated with increased mortality risk (17). Together, these findings support the clinical and public health importance of interventions that improve diet quality while also addressing behavioral confidence, perceived health, energy, sleep, and social connection.

Full Plate Living (FPL) is a free, digitally delivered nutrition education program developed by the nonprofit Ardmore Institute of Health to support gradual adoption of a whole food, plant-predominant, fiber-rich eating pattern. The program emphasizes adding more fiber-rich foods, such as fruits, vegetables, beans, legumes, and whole grains to meals participants already eat, rather than relying on calorie counting, restrictive dieting, or intensive coaching.

FPL is delivered primarily online through educational emails, self-guided modules, recipe resources, monthly workshops, and optional peer engagement through moderated online communities. Its design follows behavioral theories and evidence about lifestyle changes that focus on building confidence, supporting autonomy, encouraging small achievable changes, setting goals, receiving feedback, and having social support (18–20). The outcomes evaluated in this analysis were selected to reflect both the dietary pattern supported by FPL, including diet quality, dietary fiber, and food group estimates, and participant-reported indicators that may support lifestyle behavior change, including dietary self-efficacy, perceived energy, general health, sleep duration, and social ties. Because FPL is free, self-guided, and digitally accessible, it may be relevant as a scalable nutrition support program outside structured clinical or workplace programs, while recognizing that digital access and literacy may affect reach.

Prior FPL studies have shown favorable outcomes in structured settings. In a retrospective analysis of US employees who completed a 16-week workplace-based FPL program, participants reported higher intake of fiber-rich foods, better perceived health and energy, greater confidence in making healthy food choices and losing weight, and modest reductions in body weight and BMI (21). A later analysis of the same cohort found that increased intake of fiber-rich foods, especially fruits and vegetables, was associated with greater weight loss; nearly two-thirds of participants lost weight during the program period (22). A recent FPL case series also reported favorable cardiometabolic outcomes in selected participants (23). However, prior FPL evidence has largely come from structured workplace, cohort-based, or selected clinical settings, which differs from the self-guided digital context evaluated here.

The present study builds on prior FPL research by examining baseline-to-follow-up changes among adults participating in the digitally delivered FPL program using Diet ID derived measures of diet quality, including the Diet ID Quality Score and HEI-2015 (24). In addition to diet-quality outcomes, this analysis evaluates changes in BMI, dietary fiber intake, estimated energy intake, dietary self-efficacy, perceived energy, general health, sleep duration, and social ties. This analysis describes observed outcomes among participants with eligible paired assessments, compares them with participants without eligible follow-up records, and identifies methodological issues for future controlled studies of digital nutrition programs.

## Materials and methods

### Study design and data source

This secondary analysis used deidentified observational data from adults enrolled in the FPL program. The Diet ID/FPL dataset included records dated July 2023 through January 2026. Data were collected during routine program participation and were not originally intended for research. The dataset contained no direct identifiers, and investigators did not have access to a re-identification key.

Because participants may have accessed FPL content before completing their first Diet ID assessment, the first available Diet ID/FPL record was treated as an operational baseline rather than a true pre-intervention assessment. Follow-up was defined as the first subsequent record at least 60 days later. The 60-day threshold was selected to avoid classifying records completed close together as follow-up and to allow a minimum interval for potential dietary behavior change. Changes were therefore interpreted as baseline-to-follow-up changes in participants with eligible paired assessments rather than definitive pre-post intervention effects.

### Participants and pairing protocol

Adults were eligible for the paired analysis if they had a unique User ID and at least one Diet ID/FPL record that occurred 60 or more days after their first available record. Records were sorted chronologically. The first available record was selected as baseline, and the first subsequent record at least 60 days later was selected as follow-up. Participants without eligible follow-up records were defined as those without a subsequent Diet ID/FPL record at least 60 days after their first available record. For participants with two or more records who did not meet the 60-day follow-up criterion, first-to-last record intervals were summarized to clarify assessment timing. Complete-case paired observations were used for each outcome; therefore, analytic sample size varied by measure.

### Intervention overview

FPL is a free, self-guided, digitally delivered nutrition education program that emphasizes gradual adoption of a whole food, plant-predominant, fiber-rich dietary pattern. Program components include sequenced educational emails, digital learning modules, recipe resources, monthly workshops, and optional peer engagement through moderated social media communities. The program encourages participants to add fiber-rich whole plant foods to meals they already eat, including fruits, vegetables, beans, legumes, and cooked whole grains, rather than focusing on calorie counting, restrictive dieting, or intensive coaching. Because FPL is free and publicly accessible, participants can access it without clinic based delivery, insurance authorization, or structured enrollment through a health system; however, the present analysis did not evaluate provider referral workflows or comparative effectiveness with clinical nutrition services.

### Outcome measures

Diet quality was assessed using Diet ID, an image-based dietary assessment tool that estimates habitual dietary patterns and generates a Diet ID Quality Score and HEI-2015 score (24). The Diet ID Quality Score ranges from 1 to 10, and HEI-2015 scores range from 0 to 100, with higher scores indicating higher diet quality. Diet ID derived dietary and food group estimates were interpreted as exploratory measures of dietary pattern change.

Anthropometric outcomes included self-reported height and weight, from which BMI was calculated as kg/m^2^. Behavioral and experiential measures included confidence in making healthy food choices, confidence in losing weight, perceived energy level, general health status, sleep duration category, and social ties. Sleep duration category was coded as 1 = less than 4 h, 2 = 5 to 6 h, 3 = 7 to 8 h, and 4 = 9 or more hours per night. Exploratory dietary pattern outcomes included Diet ID derived dietary fiber intake, estimated energy intake, and selected food group estimates (24).

### Statistical analysis

Descriptive statistics were calculated for participants with eligible paired baseline and follow-up records. Within-participant changes were evaluated using paired *t* tests for continuous outcomes. Ordinal-coded outcomes were analyzed as exploratory paired comparisons after coding responses so that higher values reflected more favorable status where applicable. Effect sizes were calculated as Cohen's *d* for paired change, defined as the mean within-participant change divided by the standard deviation of the paired differences. Statistical significance was defined *a priori* as a two-sided *P* value <.05. Complete case paired observations were used for each outcome, and no imputation was performed. This approach treats ordered categories as approximate numeric scores for descriptive purposes and assumes that category spacing is sufficient for paired mean comparisons; these findings were interpreted as exploratory.

To address potential selection bias, baseline characteristics were compared between participants with eligible paired records and participants without eligible follow-up records. Continuous variables were compared using Welch two-sample *t* tests, categorical variables were compared using chi-square tests, and standardized mean differences were calculated for continuous variables.

Bonferroni-adjusted *P* values were calculated as sensitivity analyses for the Tables 2, 3 outcome families. Linear regression models examined whether follow-up interval was associated with within-participant change in Diet ID Quality Score, HEI-2015, BMI, and dietary fiber intake. Results were expressed as beta coefficients per 30 days, with Pearson correlations also calculated. Additional sensitivity analyses examined alternative minimum follow-up thresholds of 30, 60, 90, 180, and 365 days; follow-up duration strata within the 60-day paired sample; and baseline characteristics of participants with complete vs. incomplete key outcome data. These analyses were used to assess whether findings varied by the 60-day eligibility threshold, follow-up duration, or missing key outcome data. Exploratory subgroup analyses by baseline BMI category were also summarized descriptively. Program engagement could not be evaluated in dose-response analyses because the dataset did not include module completion, login frequency, workshop attendance, or other direct use measures.

## Results

### Participant flow and analytic sample

The January 2026 data included 7,077 records from 4,359 unique participants. Of the 4,359 participants, 1,800 had two or more records. Overall, 392 participants (9.0%) had eligible paired baseline and follow-up records separated by at least 60 days, while 3,967 did not have eligible paired data. The remaining 1,408 participants with two or more records did not meet the 60-day threshold. Among these 1,408 participants, first-to-last record intervals were <1 day for 991 (70.4%), 1–7 days for 115 (8.2%), 8–30 days for 184 (13.1%), and 31–59 days for 118 (8.4%). Among participants with eligible paired records, the median interval between records was 159.0 days (mean, 244.2 days; range, 60–846 days). Complete paired data were available for 352 participants for the Diet ID Quality Score and HEI-2015 outcomes and for 363 participants for BMI.

### Comparison of participants with and without eligible follow-up records

Baseline characteristics of participants with and without eligible follow-up records are shown in Table 1. The paired cohort was predominantly female (87.5%) and White (81.1%), with a mean age of 57.7 years and mean baseline BMI of 31.6 kg/m^2^. Compared with individuals without eligible follow-up records, paired-assessment participants were older and had slightly higher baseline Diet ID Quality Score, HEI-2015, and dietary fiber intake, as well as lower estimated energy intake. Gender distribution and primary reason for joining FPL also differed between groups. Other baseline measures, including BMI, behavioral outcomes, race/ethnicity, and BMI category, did not differ significantly. Overall, continuous baseline differences were small.

**Table 1:** Baseline characteristics of participants With and without eligible follow-up records.

Characteristic	Paired participants with eligible follow-up records	Participants without eligible follow-up records	*P* Value	Standardized Mean Difference (SMD)
Age, years	57.7 (12.1); *n* = 363	55.3 (13.0); *n* = 3,708	<.001	0.19
BMI, kg/m^2^	31.6 (7.9); *n* = 363	31.9 (7.9); *n* = 3,708	.452	−0.04
Diet ID Quality Score	7.20 (2.74); *n* = 375	6.73 (2.71); *n* = 3,830	.002	0.17
Healthy Eating Index-2015	73.80 (24.61); *n* = 375	69.73 (24.58); *n* = 3,830	.002	0.17
Dietary fiber, g/d	34.0 (18.8); *n* = 368	31.6 (18.0); *n* = 3,740	.017	0.14
Estimated energy intake, kcal/d	2050.0 (384.7); *n* = 368	2105.6 (406.8); *n* = 3740	.009	−0.14
Healthy food choice confidence, 0–10	6.75 (2.00); *n* = 370	6.58 (2.12); *n* = 3,801	.117	0.08
Weight-loss confidence, 0–10	4.83 (2.37); *n* = 340	4.88 (2.42); *n* = 3,498	.669	−0.02
Energy level, 1–5	2.70 (0.89); *n* = 372	2.73 (0.79); *n* = 3,811	.606	−0.03
General health, 1–5	3.10 (0.89); *n* = 372	3.07 (0.88); *n* = 3,811	.408	0.05
Sleep duration category, 1–4	2.63 (0.58); *n* = 373	2.64 (0.57); *n* = 3,818	.818	−0.01
Social ties, 1–5	3.75 (1.01); *n* = 373	3.74 (1.03); *n* = 3,814	.906	0.01
**Gender**			.**011**	
Female	343 (87.5)	3,343 (84.3)		
Male	14 (3.6)	305 (7.7)		
Not reported	35 (8.9)	319 (8.0)		
**Race/ethnicity**			.**152**	
White	318 (81.1)	3,172 (80.0)		
Black or African American	31 (7.9)	273 (6.9)		
Asian	4 (1.0)	90 (2.3)		
Multiple ethnicities/other	20 (5.1)	286 (7.2)		
Not reported	19 (4.8)	146 (3.7)		
**BMI category**			.**834**	
Underweight (<18.5)	3 (0.8)	16 (0.4)		
Normal weight (18.5–24.9)	61 (15.6)	660 (16.6)		
Overweight (25.0–29.9)	109 (27.8)	1,057 (26.6)		
Class I obesity (30.0–34.9)	91 (23.2)	880 (22.2)		
Class II obesity (35.0–39.9)	51 (13.0)	564 (14.2)		
Class III obesity (>=40.0)	48 (12.2)	531 (13.4)		
Missing/not reported	29 (7.4)	259 (6.5)		
**Primary reason joined FPL**			.**012**	
Weight loss	133 (33.9)	1,568 (39.5)		
Improve overall health	132 (33.7)	1,053 (26.5)		
Healthy eating	56 (14.3)	464 (11.7)		
Blood sugar management	27 (6.9)	306 (7.7)		
Better cholesterol	12 (3.1)	176 (4.4)		
Increase energy	2 (0.5)	66 (1.7)		
Lower blood pressure	4 (1.0)	72 (1.8)		
Other	8 (2.0)	117 (2.9)		
Not reported	18 (4.6)	145 (3.7)		

Values are mean (SD); n or No. (%), unless otherwise indicated. *P* values for continuous variables are from Welch two-sample t tests; *P* values for categorical variables are from chi-square tests for the overall variable. SMD indicates standardized mean difference and is reported for continuous variables. Percentages for categorical variables use group specific denominators, including missing/not reported categories.

### Changes in diet quality, BMI, and behavioral outcomes

Between baseline and follow-up, statistically significant improvements were observed in diet quality (Table 2). Diet ID Quality Score improved from 7.20 (SD, 2.72) to 7.91 (SD, 2.44; Cohen d = 0.26), and HEI-2015 increased from 73.87 (SD, 24.36) to 80.24 (SD, 21.50; Cohen d = 0.27). BMI decreased from 31.62 kg/m^2^ (SD, 7.92) to 31.03 kg/m^2^ (SD, 7.25), a mean change of −0.59 kg/m^2^ (Cohen d = −0.17). Effect sizes were small.

**Table 2:** Changes in diet quality, BMI, and behavioral outcomes From baseline to follow-up.

Outcome	*n*	Baseline Mean (SD)	Follow-up Mean (SD)	Mean Change	Cohen d	*P* Value	Bonferroni-adjusted *P* Value
Diet ID Quality Score (1–10)	352	7.20 (2.72)	7.91 (2.44)	+0.70	0.26	<.001	<.001
Healthy Eating Index-2015 (0–100)	352	73.87 (24.36)	80.24 (21.50)	+6.37	0.27	<.001	<.001
BMI (kg/m^2^)	363	31.62 (7.92)	31.03 (7.25)	−0.59	−0.17	.001	.012
Healthy food choice confidence (0–10)	345	6.77 (2.02)	7.10 (2.26)	+0.32	0.15	.004	.039
Weight-loss confidence (0–10)	304	4.78 (2.34)	5.46 (2.55)	+0.68	0.27	<.001	<.001
Energy level (1–5)	347	2.74 (0.88)	2.94 (0.83)	+0.20	0.27	<.001	<.001
General health (1–5)	347	3.13 (0.88)	3.29 (0.91)	+0.17	0.27	<.001	<.001
Sleep duration category (1–4)	348	2.64 (0.58)	2.69 (0.56)	+0.05	0.11	.039	.352
Social ties (1–5)	348	3.76 (1.02)	3.75 (1.04)	−0.01	−0.02	.761	1.000

BMI, body mass index; HEI, Healthy Eating Index. Paired t tests were used for baseline-to-follow-up comparisons. Ordinal-coded outcomes were analyzed as exploratory paired comparisons after coding responses so that higher values reflected more favorable status where applicable. Cohen d reflects standardized mean change using the standard deviation of paired differences. Positive values indicate improvement except for BMI, for which a negative value indicates reduction. All *P* values are two-sided; statistical significance was defined as *P* < .05. Bonferroni-adjusted *P* values were calculated as a sensitivity analysis across the nine outcomes shown in this table.

Statistically significant improvements were observed in healthy food choice confidence, weight-loss confidence, perceived energy, general health, and sleep duration category. Social ties did not change significantly. After Bonferroni adjustment, improvements in Diet ID Quality Score, HEI-2015, BMI, healthy food choice confidence, weight-loss confidence, energy level, and general health remained significant. The sleep duration category did not remain significant.

### Changes in dietary fiber and selected food group outcomes

Diet ID derived dietary fiber intake increased from 34.0 g/d at baseline to 38.9 g/d at follow-up, a mean increase of 5.0 g/d (Table 3). Food group changes were consistent with a higher-fiber, plant-predominant dietary pattern: beans/lentils, fruit, vegetables, whole grains, and nuts/seeds increased, while refined grains, sweetened beverages, red meat, processed meat, and sweets/desserts decreased. Estimated energy intake did not change significantly.

**Table 3:** Changes in dietary fiber, estimated energy intake, and food group outcomes From baseline to follow-up.

Outcome	*n*	Baseline Mean (SD)	Follow-up Mean (SD)	Mean Change	Cohen d	*P* Value	Bonferroni-adjusted *P* Value
Dietary fiber (g/d)	346	33.95 (18.74)	38.95 (19.02)	+5.00	0.28	<.001	<.001
Estimated energy intake (kcal/d)	346	2047.20 (383.50)	2054.81 (383.67)	+7.62	0.04	.478	1.000
Beans/lentils servings/d	352	0.58 (0.72)	0.70 (0.74)	+0.12	0.16	.002	.027
Fruit servings/d	352	2.60 (2.01)	3.17 (2.05)	+0.57	0.26	<.001	<.001
Vegetable servings/d	352	7.50 (4.72)	8.63 (4.79)	+1.13	0.24	<.001	<.001
Whole-grain servings/d	352	2.68 (2.26)	3.18 (2.25)	+0.50	0.21	<.001	.002
Nuts/seeds servings/d	352	1.59 (1.39)	1.93 (1.40)	+0.34	0.22	<.001	<.001
Refined-grain servings/d	352	3.24 (2.85)	2.79 (2.62)	−0.45	−0.14	.009	.111
Sweetened beverage servings/d	352	0.41 (0.92)	0.28 (0.78)	−0.12	−0.13	.019	.229
Red meat servings/d	352	1.13 (1.89)	0.85 (1.50)	−0.28	−0.13	.014	.164
Processed meat servings/d	352	0.58 (0.86)	0.40 (0.69)	−0.19	−0.21	<.001	.001
Sweets/desserts servings/d	352	2.08 (2.76)	1.47 (2.37)	−0.61	−0.20	<.001	.002

Food group variables are Diet ID derived estimates in servings/d unless otherwise noted; dietary fiber is reported in g/d, and estimated energy intake is reported in kcal/d. Outcomes were analyzed using complete case paired observations. Paired t tests were used for baseline-to-follow-up comparisons. Cohen d reflects standardized mean change using the standard deviation of paired differences. Positive values indicate an increase from baseline to follow-up; negative values indicate a decrease. All *P* values are two-sided; statistical significance was defined as *P* < .05. Bonferroni-adjusted *P* values were calculated as a sensitivity analysis across the 12 outcomes shown in this table.

Following Bonferroni adjustment, dietary fiber, beans/lentils, fruit, vegetables, whole grains, nuts/seeds, processed meat, and sweets/desserts remained significant. Refined grains, sweetened beverages, and red meat did not remain significant after adjustment.

### Association between follow-up interval and outcome change

Follow-up interval was weakly associated with smaller improvements in HEI-2015 (*β* per 30 days, −0.44; *P* = .025; R^2^ = 0.014) and dietary fiber intake (beta per 30 days, −0.42 g/d; *P* = .006; R^2^ = 0.022). Follow-up interval was not associated with BMI change (*P* = .895). Diet ID Quality Score showed a similar inverse direction, but the association did not reach statistical significance (beta per 30 days, −0.04; *P* = .052). Although Diet ID Quality Score showed a similar inverse direction, Table 4 shows that the association did not reach statistical significance (*β* per 30 days, −0.04; *P* = .052).

**Table 4:** Association between follow-up interval and change in Key outcomes.

Outcome	*n*	Pearson r	*P* Value	*β* per 30 days	95% CI	R^2^
Diet ID Quality Score	352	−0.104	.052	−0.04	−0.09 to 0.00	0.011
Healthy Eating Index-2015	352	−0.119	.025	−0.44	−0.83 to −0.06	0.014
BMI	363	0.007	.895	0.00	−0.05 to 0.06	0.000
Dietary fiber	346	−0.149	.006	−0.42	−0.71 to −0.12	0.022

Linear regression models evaluated the association between days between baseline and follow-up records and within-participant change in key outcomes. Beta coefficients (β) represent change in the outcome per 30 additional days between assessments. CI, confidence interval.

### Additional sensitivity analyses

Supplementary Table 1 summarizes the additional analyses. Using 30-, 60-, 90-, 180-, and 365-day follow-up thresholds, Diet ID Quality Score, HEI-2015, BMI, and dietary fiber intake changed in the same general direction as the primary analysis. Effect sizes remained small and were generally smaller at longer thresholds. In follow-up duration strata within the 60-day paired sample, dietary fiber change differed across strata, with the largest increase in the 60–90 day group; between-strata differences were not significant for Diet ID Quality Score, HEI-2015, or BMI. Among the 392 participants with eligible paired records, 343 (87.5%) had complete paired data for all four key outcomes. Baseline continuous differences between participants with complete and incomplete key outcome data were generally small. Exploratory baseline BMI subgroup analyses suggested larger diet-quality and BMI changes among participants with class II/III obesity, but these subgroup findings were descriptive, not prespecified, and should be interpreted cautiously.

## Discussion

### Principal findings

In this exploratory secondary analysis of routine digital program data, the 9.0% of participants with eligible paired assessments had baseline-to-follow-up changes in diet quality, BMI, dietary self-efficacy, perceived energy, general health, dietary fiber intake, and selected food group outcomes. The strongest and most directly relevant findings were improvements in diet quality and dietary fiber intake. However, effect sizes were small, the analytic sample was highly selected, and the findings should be interpreted as hypothesis-generating rather than evidence of program effectiveness in all FPL participants.

Baseline dietary fiber intake was relatively high, and paired-assessment participants had higher baseline diet quality and fiber intake than participants without eligible follow-up records. This pattern supports the interpretation that the analytic sample may have been more health-conscious or more engaged at baseline. BMI decreased by 0.59 kg/m^2^, although the standardized effect size was small and the clinical significance is uncertain. Because estimated energy intake did not change significantly and both weight and dietary intake were self-reported or Diet ID derived, BMI and energy-intake findings should be interpreted cautiously. The BMI change could also reflect unmeasured physical activity, concurrent interventions, medication changes, regression to the mean, random within-person variation, or other lifestyle changes rather than measured dietary change alone.

### Prior FPL and fiber research

Prior FPL studies in structured workplace settings reported favorable changes in fiber-rich food intake, perceived health, confidence, and weight-related outcomes (21, 22). The current analysis differs because it evaluated routine data from a self-guided digital program with variable exposure, no standardized engagement measure, and no control group. Therefore, these findings should be viewed as a descriptive extension of prior FPL work rather than a direct test of comparative effectiveness.

Findings from this analysis are also consistent with broader fiber-focused nutrition studies in workplace and community settings, while showing smaller effects than might be expected in more structured programs (25–27). The smaller BMI change is not unexpected given the self-guided digital format and lack of measured program dose. Follow-up time alone did not explain the modest BMI reduction.

### Behavioral and participant-reported outcomes

Participants reported improvements in healthy food choice confidence, weight-loss confidence, perceived energy, general health, and sleep duration category. After Bonferroni adjustment, healthy food choice confidence, weight-loss confidence, perceived energy, and general health remained significant. These participant-reported outcomes may reflect proximal changes that support self-directed behavior change, but they were assessed using ordinal-coded or single-item measures and should be interpreted as exploratory.

Sleep duration category improved modestly in the unadjusted analysis but did not remain significant after Bonferroni adjustment. Sleep was assessed categorically rather than with a validated sleep quality instrument. Social ties did not change significantly, which may reflect that peer engagement in the digital FPL program is optional, that exposure to social components was not measured, or that the social ties measure did not capture program-specific support.

Additional sensitivity analyses supported a cautious interpretation. Direction of change was generally consistent across alternative follow-up thresholds, but effect sizes remained small. Follow-up duration strata suggested larger dietary fiber changes at shorter follow-up intervals and smaller changes at longer intervals, whereas BMI change did not differ meaningfully by follow-up duration. Future work should evaluate whether actual program engagement, rather than elapsed time alone, better explains dietary pattern change.

### Implications for primary care and lifestyle medicine

These findings may inform future evaluation of FPL as a potential digital nutrition resource in clinical and community settings. However, the present study did not evaluate provider referral workflows, provider attitudes, patient adherence to referrals, or comparative effectiveness with existing referral options such as individualized medical nutrition therapy or registered dietitian nutritionist care. Therefore, the findings should not be interpreted as evidence that FPL is an effective referral intervention.

FPL is available at no cost to participants, but accessibility is not universal. Use of a digital program requires internet access, digital literacy, time, and likely English-language proficiency. These equity and access considerations should be addressed in future implementation studies before drawing conclusions about reach across diverse clinical or community populations.

### Limitations

Several limitations should be considered. This observational analysis did not include a control group; therefore, observed changes cannot be attributed to FPL. The first available Diet ID/FPL record was used as the operational baseline and may not reflect program enrollment or preprogram dietary behavior, particularly for participants who accessed FPL before completing their first assessment. Only 392 of 4,359 participants (9.0%) had eligible paired assessments; therefore, findings apply to a selected subset of participants who completed repeat Diet ID assessments and not to all FPL participants. Because the paired sample was defined using a 60-day minimum follow-up threshold, this eligibility criterion may have influenced both sample composition and the magnitude of observed changes, although sensitivity analyses using alternative thresholds showed generally consistent direction of findings.

Selection bias and missing data are important concerns. Paired-assessment participants differed from those without eligible follow-up records on several baseline characteristics. Although continuous baseline differences were generally small, the analytic sample may represent a more engaged or diet-quality-oriented subset of participants, and missing data may not have been random. Complete case analyses were conducted by outcome; additional analyses suggested small continuous baseline differences between participants with complete and incomplete key outcome data, but missing demographic and categorical data remained a limitation.

Generalizability is limited because the paired cohort was predominantly female, White, and older. Participants also reported relatively high dietary fiber intake at baseline.

Outcomes were self-reported or Diet ID derived and may be affected by reporting, measurement, or social desirability bias. Diet ID derived HEI-2015 and food group estimates should be interpreted in light of the dietary assessment method and its validation limitations in this specific population. Validation evidence for Diet ID may not fully generalize to this selected cohort, and systematic differences in how participants completed baseline and follow-up assessments could contribute to observed dietary changes. Sleep duration and social ties were single-item measures, and no validated sleep quality or social support instrument was used. The analysis also lacked direct engagement or dose measures and did not evaluate provider referral workflows, digital access, language, literacy, or cultural appropriateness. Unmeasured confounding, including physical activity, concurrent interventions, health care utilization, medication changes, and other lifestyle changes, may also have contributed to observed changes. Secondary and exploratory findings should be interpreted cautiously, particularly when effect sizes were small. Potential conflicts related to author affiliations and funding from the program operator are disclosed separately.

### Future directions

Future studies should use controlled or pragmatic designs with prespecified follow-up intervals, stronger engagement and dose measures, and objective clinical outcomes such as measured weight, waist circumference, blood pressure, lipid levels, glycemic markers, and medication changes. Future work should also evaluate implementation in primary care and community referral workflows, including provider adoption, participant adherence, comparative effectiveness with existing nutrition referral options, digital access, language, cultural relevance, and equity across diverse populations. Future studies should also include validated measures of dietary self-efficacy, sleep quality, social support, and participant experience.

## Conclusion

This exploratory secondary analysis found that the 9.0% of FPL participants with eligible paired assessments had small favorable baseline-to-follow-up changes in diet quality, BMI, dietary self-efficacy, perceived energy, general health, dietary fiber intake, and selected food group outcomes. These findings are limited to participants with paired data and should be interpreted as hypothesis-generating rather than causal evidence of program effectiveness. Controlled studies with stronger engagement measures, objective clinical outcomes, more complete follow-up, and evaluation of clinical or community referral workflows are warranted.

## Data Availability

The raw data supporting the conclusions of this article will be made available by the authors, without undue reservation.

## Supplementary material

SUPPLEMENTARY TABLE 1 Additional sensitivity analyses.
