# Supplementary material for: Digitally delivered full plate living: associations with diet quality, body mass index, and participant-reported health measures among participants with paired assessments
Source: Front Digit Health. 2026 Oct 12;8:1947833. doi: 10.3389/fdgth.2026.1947833 (PMC13696483; doi:10.3389/fdgth.2026.1947833)
Supplement: Supplementary file 1 [file Table1.docx]

Supplementary Table 1. Additional Sensitivity Analyses

Panel A. Alternative Minimum Follow-up Thresholds

| **Threshold** | **Eligible participants** | **Median follow-up days** | **Outcome** | **n** | **Baseline mean** | **Follow-up mean** | **Mean change** | **Cohen d** | **Paired P value** |
| --- | --- | --- | --- | --- | --- | --- | --- | --- | --- |
| ≥30 days | 510 | 96.4 | Diet ID Quality Score | 462 | 7.21 | 7.99 | 0.79 | 0.28 | <.001 |
| ≥30 days | 510 | 96.4 | Healthy Eating Index-2015 | 462 | 73.95 | 80.84 | 6.88 | 0.28 | <.001 |
| ≥30 days | 510 | 96.4 | BMI | 473 | 31.71 | 31.16 | -0.55 | -0.18 | <.001 |
| ≥30 days | 510 | 96.4 | Dietary fiber (g/d) | 454 | 33.99 | 39.65 | 5.66 | 0.30 | <.001 |
| ≥60 days | 392 | 159.0 | Diet ID Quality Score | 352 | 7.20 | 7.91 | 0.70 | 0.26 | <.001 |
| ≥60 days | 392 | 159.0 | Healthy Eating Index-2015 | 352 | 73.87 | 80.24 | 6.37 | 0.27 | <.001 |
| ≥60 days | 392 | 159.0 | BMI | 363 | 31.62 | 31.03 | -0.59 | -0.17 | .001 |
| ≥60 days | 392 | 159.0 | Dietary fiber (g/d) | 346 | 33.95 | 38.95 | 5.00 | 0.28 | <.001 |
| ≥90 days | 314 | 225.0 | Diet ID Quality Score | 281 | 7.22 | 7.78 | 0.56 | 0.22 | <.001 |
| ≥90 days | 314 | 225.0 | Healthy Eating Index-2015 | 281 | 73.92 | 78.94 | 5.01 | 0.22 | <.001 |
| ≥90 days | 314 | 225.0 | BMI | 291 | 31.40 | 30.83 | -0.56 | -0.15 | .010 |
| ≥90 days | 314 | 225.0 | Dietary fiber (g/d) | 277 | 34.09 | 37.44 | 3.35 | 0.20 | .001 |
| ≥180 days | 205 | 380.0 | Diet ID Quality Score | 185 | 7.39 | 7.84 | 0.45 | 0.18 | .013 |
| ≥180 days | 205 | 380.0 | Healthy Eating Index-2015 | 185 | 75.43 | 79.28 | 3.85 | 0.18 | .018 |
| ≥180 days | 205 | 380.0 | BMI | 194 | 31.21 | 30.80 | -0.41 | -0.17 | .017 |
| ≥180 days | 205 | 380.0 | Dietary fiber (g/d) | 184 | 35.72 | 38.15 | 2.43 | 0.15 | .042 |
| ≥365 days | 120 | 521.2 | Diet ID Quality Score | 107 | 7.44 | 7.96 | 0.52 | 0.22 | .028 |
| ≥365 days | 120 | 521.2 | Healthy Eating Index-2015 | 107 | 75.81 | 80.09 | 4.28 | 0.20 | .043 |
| ≥365 days | 120 | 521.2 | BMI | 113 | 31.83 | 31.25 | -0.58 | -0.21 | .025 |
| ≥365 days | 120 | 521.2 | Dietary fiber (g/d) | 107 | 36.85 | 39.20 | 2.34 | 0.14 | .137 |

Note. All analyses used the January 2026 Diet ID/FPL dataset. Follow-up was defined as the first subsequent record at or beyond the specified minimum threshold. P values reflect within-participant baseline-to-follow-up paired comparisons within each threshold and were not adjusted for multiple testing. Cohen d reflects standardized mean change using the standard deviation of paired differences. These analyses were conducted as sensitivity analyses rather than prespecified primary analyses; findings were generally consistent across thresholds, although effect sizes remained small and dietary fiber change attenuated at longer thresholds.

Panel B. Outcomes stratified by follow-up duration among participants with 60-day paired records

| **Outcome** | **Stratum** | **Eligible participants** | **n** | **Median days** | **Mean change** | **Cohen d** | **Within-stratum P value** | **Between-strata P value** |
| --- | --- | --- | --- | --- | --- | --- | --- | --- |
| Diet ID Quality Score | 60-90 days | 88 | 81 | 74.0 | 1.12 | 0.37 | .001 | .186 |
| Diet ID Quality Score | 91-180 days | 123 | 108 | 113.9 | 0.76 | 0.28 | .004 | - |
| Diet ID Quality Score | 181+ days | 181 | 163 | 374.0 | 0.46 | 0.19 | .018 | - |
| Healthy Eating Index-2015 | 60-90 days | 88 | 81 | 74.0 | 10.30 | 0.39 | <.001 | .146 |
| Healthy Eating Index-2015 | 91-180 days | 123 | 108 | 113.9 | 6.95 | 0.29 | .003 | - |
| Healthy Eating Index-2015 | 181+ days | 181 | 163 | 374.0 | 4.04 | 0.18 | .020 | - |
| BMI | 60-90 days | 88 | 81 | 74.0 | -0.65 | -0.27 | .018 | .597 |
| BMI | 91-180 days | 123 | 111 | 113.9 | -0.83 | -0.16 | .090 | - |
| BMI | 181+ days | 181 | 171 | 374.0 | -0.41 | -0.17 | .031 | - |
| Dietary fiber (g/d) | 60-90 days | 88 | 79 | 74.0 | 9.35 | 0.43 | <.001 | .014 |
| Dietary fiber (g/d) | 91-180 days | 123 | 105 | 113.9 | 5.92 | 0.32 | .001 | - |
| Dietary fiber (g/d) | 181+ days | 181 | 162 | 374.0 | 2.28 | 0.15 | .063 | - |

Note. The P value column reflects within-stratum baseline-to-follow-up change. Between-strata P values test whether mean change differed across follow-up-duration strata for each outcome. Dietary fiber change differed across strata; changes in Diet ID Quality Score, HEI-2015, and BMI did not differ significantly across strata.

Panel C. Baseline continuous characteristics among participants with complete versus incomplete paired key outcomes

| **Characteristic** | **Complete n** | **Complete mean (SD)** | **Incomplete n** | **Incomplete mean (SD)** | **P value** | **SMD** |
| --- | --- | --- | --- | --- | --- | --- |
| Age, years | 343 | 57.73 (12.14) | 20 | 58.05 (11.31) | .902 | -0.03 |
| BMI, kg/m² | 343 | 31.62 (7.95) | 20 | 31.60 (7.65) | .990 | 0.00 |
| Diet ID Quality Score | 343 | 7.19 (2.73) | 32 | 7.31 (2.93) | .817 | -0.04 |
| HEI-2015 | 343 | 73.69 (24.44) | 32 | 74.94 (26.74) | .801 | -0.05 |
| Dietary fiber, g/d | 343 | 33.97 (18.82) | 25 | 34.73 (19.57) | .851 | -0.04 |
| Estimated energy intake, kcal/d | 343 | 2047.76 (385.13) | 25 | 2080.49 (385.56) | .685 | -0.08 |
| Healthy food choice confidence | 343 | 6.78 (1.99) | 27 | 6.37 (2.10) | .336 | 0.20 |
| Weight-loss confidence | 315 | 4.80 (2.32) | 25 | 5.12 (3.03) | .614 | -0.12 |
| Energy level | 343 | 2.72 (0.85) | 29 | 2.52 (1.21) | .385 | 0.19 |
| General health | 343 | 3.12 (0.88) | 29 | 2.97 (0.94) | .412 | 0.17 |
| Sleep duration category | 343 | 2.64 (0.58) | 30 | 2.53 (0.57) | .341 | 0.18 |
| Social ties | 343 | 3.75 (1.02) | 30 | 3.73 (0.87) | .939 | 0.01 |

Note: Complete key paired outcomes were defined as complete baseline and follow-up values for Diet ID Quality Score, HEI-2015, BMI, and dietary fiber. A total of 343 participants had complete key paired outcomes, and 49 were incomplete for at least one key paired outcome. P values compare baseline characteristics between complete and incomplete groups. SMD indicates standardized mean difference. Continuous baseline differences were generally small.

**Panel D. Exploratory baseline BMI subgroup analysis**

| **BMI subgroup** | **Outcome** | **Eligible participants** | **n** | **Baseline mean** | **Follow-up mean** | **Mean change** | **Within-subgroup P value** | **Between-subgroup P value** |
| --- | --- | --- | --- | --- | --- | --- | --- | --- |
| Normal/underweight | Diet ID Quality Score | 64 | 62 | 8.77 | 8.84 | 0.06 | .830 | .004 |
| Overweight | Diet ID Quality Score | 109 | 103 | 7.29 | 8.12 | 0.83 | .003 | - |
| Class I obesity | Diet ID Quality Score | 91 | 85 | 7.39 | 7.71 | 0.32 | .221 | - |
| Class II/III obesity | Diet ID Quality Score | 99 | 93 | 5.83 | 7.30 | 1.47 | <.001 | - |
| Normal/underweight | Healthy Eating Index-2015 | 64 | 62 | 87.34 | 88.40 | 1.06 | .684 | .009 |
| Overweight | Healthy Eating Index-2015 | 109 | 103 | 74.70 | 81.50 | 6.81 | .005 | - |
| Class I obesity | Healthy Eating Index-2015 | 91 | 85 | 75.55 | 79.14 | 3.59 | .119 | - |
| Class II/III obesity | Healthy Eating Index-2015 | 99 | 93 | 61.78 | 74.81 | 13.02 | <.001 | - |
| Normal/underweight | BMI | 64 | 64 | 22.40 | 22.36 | -0.04 | .785 | .001 |
| Overweight | BMI | 109 | 109 | 27.55 | 27.49 | -0.06 | .791 | - |
| Class I obesity | BMI | 91 | 91 | 32.22 | 31.86 | -0.36 | .083 | - |
| Class II/III obesity | BMI | 99 | 99 | 41.50 | 39.75 | -1.75 | .003 | - |
| Normal/underweight | Dietary fiber (g/d) | 64 | 62 | 40.85 | 42.13 | 1.28 | .502 | .029 |
| Overweight | Dietary fiber (g/d) | 109 | 103 | 33.38 | 39.85 | 6.47 | <.001 | - |
| Class I obesity | Dietary fiber (g/d) | 91 | 85 | 33.08 | 35.29 | 2.21 | .229 | - |
| Class II/III obesity | Dietary fiber (g/d) | 99 | 93 | 30.83 | 39.47 | 8.64 | <.001 | - |

Note. Subgroup analyses were exploratory and not prespecified. The P value column reflects within-subgroup baseline-to-follow-up change. The between-subgroup P value compares mean change across BMI subgroups for each outcome. Sample sizes vary by outcome; results should not be interpreted as confirmatory tests of effect modification.
